# Tricaine Methanesulfonate (MS222) Has Short-Term Effects on Young Adult Zebrafish (*Danio rerio*) Working Memory and Cognitive Flexibility, but Not on Aging Fish

**DOI:** 10.3389/fnbeh.2021.686102

**Published:** 2021-08-04

**Authors:** Barbara D. Fontana, Nancy Alnassar, Matthew O. Parker

**Affiliations:** Brain and Behaviour Laboratory, School of Pharmacy and Biomedical Sciences, University of Portsmouth, Portsmouth, United Kingdom

**Keywords:** working memory, MS222, cognition, FMP *Y*-maze, behavioral flexibility, zebrafish

## Abstract

Exposure to anesthetic drugs is common in biomedical sciences being part of routine procedures in different translational species, however its impacts on memory and cognition are still debated, having different impacts depending on drug and age. The zebrafish (*Danio rerio*) is a translational species widely used in behavioral neuroscience, where tricaine methanesulfonate (MS222) is the most acceptable and used drug when conducting routine procedures. Based on this, we investigated the effects of MS222 (100 mg/l) in young adults and aging zebrafish 1, 2, 3, and 7 days after exposure. Animals’ were submitted to the anesthetic procedure until loss of body posture, slowing of opercular movements and lack of response to tail touch with a plastic pipette were achieved, then further left in the drug for 3 min. After that, animals (6 mpf vs. 24 mpf) were transferred to a recovery tank until fully recovered and transferred back to their housing system until further testing in the free movement pattern (FMP) *Y*-maze, which assesses zebrafish working memory and cognitive lexibility. Young animals had significant impairment in their working memory and cognitive flexibility 1 and 2 days after the exposure to MS222, being fully recovered by day 3 and with no effects 7 days post drug exposure. Increased repetitions were also observed for animals exposed to MS222 which could indicate increased stress-related response in animals up to 2 days after drug exposure. No drug effect was observed in aging animals besides their natural decreased alternations and working memory. Overall, behavioral experiments after routine procedures using MS222 should be performed with caution and need to be delayed, at least 3 days after exposure where working memory, cognitive flexibility, and repetitive behavior are back to normal.

## Introduction

Anesthesia is a common procedure used to decrease/avoid pain and distress in both clinics and in a research context (Mao et al., 1992; Gerges et al., 2006; Sobanko et al., 2012; Stewart et al., 2015). Anesthetics are often linked to the development of delayed cognitive recovery in patients that were under general anesthesia (Frazier and Narahashi, 1975; Caza et al., 2008). These drugs are often associated with neurotoxicity and have different impacts on the brain, depending on the anesthetic (Soriano and Anand, 2005; Nikizad et al., 2007; Leung et al., 2014). The intensity and patterns of cognitive disruption have been previously linked to an individual’s age (Moller et al., 1998; Culley et al., 2003). For example, a study comparing young and aging rats observed an increase in cognition after anesthesia in young rats (6 months old); meanwhile aging rats had a significant decrease in memory and cognition having long–lasting effects for up to 3 weeks after anesthesia (Culley et al., 2003). In elderly people, cognitive impairment post-operation also has long–lasting effects affecting patients’ lives up to 3 months after their surgery (Moller et al., 1998). However, there are still conflicting data regarding the long–lasting cognitive effects of anesthetics where data variability seems to be highly influenced by age of animals, choice of anesthetic drug, dose, duration of exposure, and choice of cognitive task (Belrose and Noppens, 2019).

Zebrafish (*Danio rerio*) are widely used as a translational model to study memory and cognition (Best and Alderton, 2008; Meshalkina et al., 2017; Fontana et al., 2018), owing to homologous neurotransmitter-related systems and brain regions necessary to assess these behavioral domains (Stewart et al., 2015). Tricaine methanesulfonate (MS222) is an anesthetic drug used widely across the zebrafish research community (Stewart et al., 2015). This anesthetic is often used during routine husbandry procedures such as “*fin-clipping*” (removal of the tip of the caudal fin, to collect samples for genotypic identification; Huang et al., 2010; Xing et al., 2014; De Lombaert et al., 2017). MS222 induces anesthesia by blocking sodium channels in the neuronal membranes, thus reducing action potentials and inducing muscle relaxation (Frazier and Narahashi, 1975; Matthews and Varga, 2012; Lin et al., 2016). In fish species, MS222 is known for crossing the blood-brain barrier (Hunn, 1970) blocking the action potential generation in sensory and motor systems as well as in central nervous circuits (Ramlochansingh et al., 2014). Because sodium channel blockers can decrease working memory in young mice by causing deleterious effects on the prelimbic cortex (Vandesquille et al., 2013), the exposure to MS222 in different vertebrate species may alter cognitive-related parameters such as working memory and cognitive flexibility. Although cognitive flexibility and working memory are both important characteristics that help different species to perform complex problem-solving tasks, these two functions are differently defined (Ionescu, 2012; Cowan, 2017). Cognitive flexibility is often defined as the adaptability to changing task conditions (Ionescu, 2012), meanwhile working memory is defined as short-term memory applied to cognitive tasks (Cowan, 2008). Currently, the short-term cognitive effects of MS222 exposure in zebrafish are unknown. It is critical to understand this in order to ensure that researchers mitigate the potential short and longer–term impacts of using this drug.

Recently, zebrafish have also shown great promise as a model to study the cognitive effects of aging (Yu et al., 2006). For example, aged zebrafish have impaired working memory and lower cognitive flexibility compared to adult animals (Cleal et al., 2021a). Typically, zebrafish changes in cognition associated with aging are evident from around 2 years old, with a gradual cognitive decline observed until 3 years old (estimated life span; Tsai et al., 2007). As well as showing cognitive decline, aging animals have been shown to respond differentially to drug treatments, including anesthetics and sedative drugs (Culley et al., 2003; Butterfield et al., 2004). The impact of MS222 on the cognitive abilities of aging zebrafish, however, is also currently not known. Owing to the increasing interest in zebrafish as a model of healthy aging, and the use of anesthetics and sedatives for routine procedures (e.g., genetic identification), it is critical to understand how these drugs affect aging zebrafish.

In summary, although the concentration required to induce anesthesia is known (Huang et al., 2010; Zellar et al., 2018), the long–lasting effects of MS222 on cognition are poorly understood, and the understanding about the effects on aged zebrafish is very limited. Here, we investigated the long–lasting effects of the anesthetic MS222 in adult (6 months old) and aging (24 months old) zebrafish using the free movement pattern (FMP) *Y*-maze task, a protocol that allowed us to explore working memory and cognitive flexibility.

## Materials and Methods

### Animals and Husbandry

Adult zebrafish (AB wild-type; ~50:50 male:female ratio at 6-month or 24-month post-fertilization) were bred in-house and reared in standard laboratory conditions on a re-circulating system (Aquaneering, USA). Animals were kept in groups of 10 per 2.8 L (~28.5°C (±1 °C); pH 8.4; 14/10-h light/dark cycle) and fed three times a day with a mixture of live brine shrimp and flake flood. All animals used for this experiment were euthanized using 2-phenoxyethanol from Aqua-Sed (Aqua-Sed^TM^, Vetark, Winchester, UK). All experiments were carried out following approval from the University of Portsmouth Animal Welfare and Ethical Review Board, and under license from the UK Home Office [Animals (Scientific Procedures) Act, 1986; PPL: P9D87106F].

### Experimental Design and MS222 Treatment

There were two factors: Factor 1 (2 levels)—age [6 vs. 24 months post-fertilization (mpf)]; and factor 2 (5 levels)—period of time after exposure to MS222 (Controls, 1, 2, 3, and 7 days). Control animals were exposed to home tank water having similar handling as MS222 groups; however, controls were randomly tested during 1, 2, 3, or 7 days after handling. Animals were exposed to MS222 at a concentration of 100 mg/l buffered with sodium bicarbonate (pH = 7.5; Grush et al., 2004; Martins et al., 2016) and fish were kept in the solution for 3 min after loss of body posture, slowing opercular movements and lack of response to tail touch with a plastic pipette (Huang et al., 2010; Zellar et al., 2018). The method was chosen considering variations in weight and size that can affect how fast animals can have a loss of their body posture and no-touch reflex. Animals were then moved to a recovery tank until equilibrium was regained and normal body movements (preanesthetic appearance) were obtained before being transferred to their home tanks. After recovery, their cognitive and memory-related effects were assessed using the FMP *Y*-maze protocol (Cleal and Parker, 2018; Fontana et al., 2019; Cleal et al., 2021a, b) by evaluating their behavioral profile 1, 2, 3, or 7 days after handling/MS222 exposure.

### FMP *Y*-Maze: Working Memory and Cognitive Flexibility Analysis

The FMP *Y*-maze is a well-characterized task to assess working memory and cognitive flexibility in zebrafish and other translational species (Cleal and Parker, 2018; Fontana et al., 2019; Cleal et al., 2021a, b). This task has been validated as a measure of working memory, with several memory-related drugs (scopolamine, MK-801, and SCH-23390), significantly affecting fishes predominant searching strategy, sequential alternations (Cleal et al., 2021b). Briefly, the apparatus used was a white Y-shaped insert with three identical arms (5 cm × 2 cm l × W; 120° angle between arms) with no explicit intra-maze cues, containing 3l of aquarium water. The Zantiks AD system (Zantiks Ltd., Cambridge, UK) was used to measure the fish choice for each arm across 1-h and their behavioral phenotype was analyzed considering the overlapping series of four choices (tetragrams; Cleal et al., 2021b). One hundred and seventy zebrafish were used to assess how MS222 affects cognition in adults vs. aging animals. The required sample size was calculated *a priori* [effect size (d) = 0.25, power = 0.99, alpha = 0.05] based on several published articles using zebrafish in the FMP *Y*-maze task and extensive experience with this protocol in our laboratory (Cleal and Parker, 2018; Fontana et al., 2019; Cleal et al., 2021a, b; Fontana et al., 2021).

Data were split into two formats for analysis: (1) total percentage use (calculated as a proportion of total turns) of each tetragram sequence for the 60 min of exploration, referred to as “global” search strategy; (2) “immediate” search strategy, analyzed search pattern configurations over 10 min time bins throughout the trial, equating to six equal, consecutive time bins. “Global” search strategy was used to measure working memory as repetition of previous turn choices must be remembered for patterns of movement to be repeated over 1 h of exploration, requiring memory of arm entries and order of entry. The second type of strategy was used to assess cognitive flexibility. Animals that are not able to update information gained during exploration of the FMP *Y*-maze will likely perform similar strategies over each time bin. However, animals that adapt their behavioral response to new information would be expected to change movement patterns over time. For example, control animals show a peak in alternation patterns at 40 min time bin based on previous characterization studies; meanwhile, animals exposed to drugs that affect cognitive flexibility do not show changes in alternations across time. Thus, to assess “global” strategies, the number of alternations (rlrl + lrlr) and repetitions (rrrr + llll) were used as proportion of the total number of turns which are highly expressed through 1-h (% of total turns; Cleal et al., 2021b). Average turns were used to assess animals’ locomotor/exploratory behavior. Alternations and repetitions across time were used to assess cognitive flexibility.

All behavioral tests were performed between 10:00 and 16:00 h (lights on at 9:00 h) using two independent batches (*n* = 8–9 per batch/per group, total *n* = 15–16/per group). The behavioral testing was carried out in a fully randomized order, choosing fish at random from one of five housing tanks for testing. After data collection and screening for extreme outliers (e.g., returning values of “0” for arm exploration indicating non-engagement), data was analyzed in full. A total of 10 animals out of 170 were excluded due to poor engagement with the task (freezing or/and displaying no measurable behavioral patterns).

### Statistics

Data from the *Y*-maze protocol was obtained as the number of entries into each arm (1, 2, 3, and middle section 4) across a 1-h trial. To analyze the data according to left and right turns in 10-min time bins, raw data was processed using the Zantiks *Y*-maze Analysis Script created specifically for this purpose (available from: https://github. com/thejamesclay/ZANTIKS_YMazeAnalysisScript). Normality of data and homogeneity of variance were analyzed by Kolmogorov–Smirnov and Bartlett’s tests, respectively. Considering that data were normally distributed, one-way analysis of covariance (ANOVA) with treatment (five levels—control vs. 1 d MS222 vs. 2 d MS222 vs. 3 d MS222 vs. 7 d MS222 for each age group) as the factor, was used to analyze average turns, repetitions, and alternations of each age. Two-way repeated measures ANOVA was used for cognitive flexibility analysis using time (six levels: 6 × 10 min time bins) and treatment (five levels: control vs. 1 d MS222 vs. 2 d MS222 vs. 3 d MS222 vs. 7 d MS222) as factors. For comparison of average turns, alternations, and repetitions between ages two-way ANOVA was performed using treatment (five levels—control vs. 1 d MS222 vs. 2 d MS222 vs. 3 d MS222 vs. 7 d MS222) and age (two levels—6 mpf vs. 24 mpf) as factors. Tukey’s test was used as *post hoc* analysis, data was represented as mean and error of the mean (SEM), and results were considered significant when *p* ≤ 0.05.

## Results

### Short and Long-Term Effects of MS222 in Adult Zebrafish

**Figure 1:**
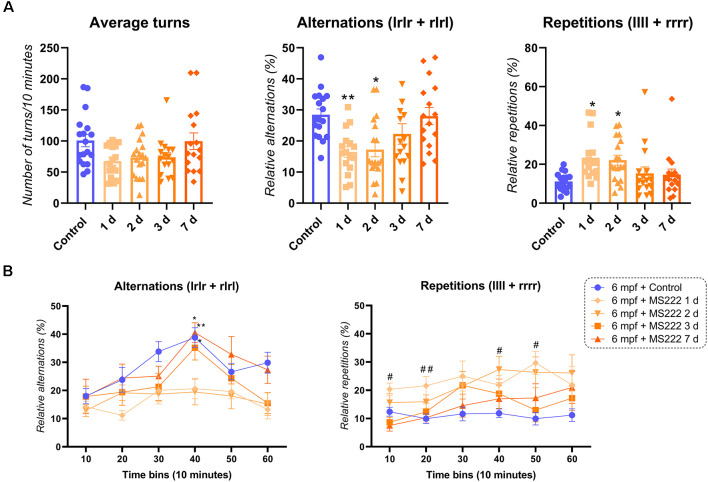
FMP *Y*-maze response of 6 months post-fertilization (mpf) animals 1, 2, 3, or 7 days after exposure to MS222. **(A)** Overall behavioral patterns/10 min. **(B)** Alternations and repetitions across time for 6 mpf controls vs. 1 day vs. 2 days vs. 3 days vs. 7 days after MS222 exposure. Asterisk represents differences across time within groups (p* < 0.05; p** < 0.005) and octothorp represents differences between groups at a specific time bin (*p*^#^ < 0.05; *p*^##^ < 0.005). Data were represented as mean ± S.E.M and analyzed by one-way ANOVA or two-way repeated measures ANOVA, followed by Tukey’s test multiple comparison test (*n* = 16–18 per group).

Figure 1 depicts the effects of MS222 in 6 mpf zebrafish. There was a significant ANOVA effect of MS222 in 6 mpf animals for alternations (*F*_(4, 79)_ = 5.556; p*** = 0.0005), repetitions (*F*_(4, 79)_ = 4.067; p*** = 0.0255). Tukey’s *post hoc* test revealed that animals had a significant decrease in alternations 1 d (p*** = 0.0066) and 2 d (p*** = 0.0095) after the exposure to MS222 compared to controls but had fully recovered after 3 d (*p* = 0.3710) and 7 d (*p* > 0.9999). For repetitions, a significant increase was observed for 1 d (p*** = 0.0117) and 2 d (p*** = 0.0240) after MS222 exposure compared to controls. No *post hoc* effects were observed for average turns after Tukey’s test analysis (Figure 1A). Regarding cognitive flexibility, there was a significant effect for time (*F*_(3.278, 259)_ = 11.05; p**** < 0.0001), treatment (*F*_(4, 79)_ = 5.566; p*** = 0.0005) and subject (*F*_(79, 395)_ = 3.028; p*** < 0.0001). No interaction effect (time vs. treatment; *F*_(20, 395)_ = 0.3700; *p* = 1.261) was observed for alternations across time. Briefly, both groups control, 3 d and 7 d after MS222 had a peak in their alternations at 40 min compared to their 10 min time bin (p*** = 0.0037, p*** = 0.0103 and p*** = 0.0175, respectively). When comparing differences between groups, a significant decrease in alternations was specifically observed when comparing 1 d and 2 d vs. controls at 40 min (p*** = 0.0086 and p*** = 0.0164; Figure 1B). A treatment (*F*_(4, 79)_ = 4.067; p*** = 0.0047), time (*F*_(3.141, 248.2)_ = 4.067; p*** = 0.0031) and subject effect (*F*_(79, 395)_ = 4.249; p^****^ < 0.0001) was also observed for repetitions across time with no interaction (time vs. treatment) effect (*F*_(20, 395)_ = 1.122; *p* = 0.3235). A significant increase in repetitions was observed 1 day after MS222 compared to controls at 10 min (p*** = 0.0481), 20 min (p*** = 0.0350) and 50 min (p*** = 0.0022). Repetitions were also increased for the group 6 mpf + 2 d MS222 at 40 min when compared to controls (p*** = 0.0357; Figure 1B).

### MS222 Does Not Affect Working Memory and Cognitive Flexibility of Aging Fish

**Figure 2:**
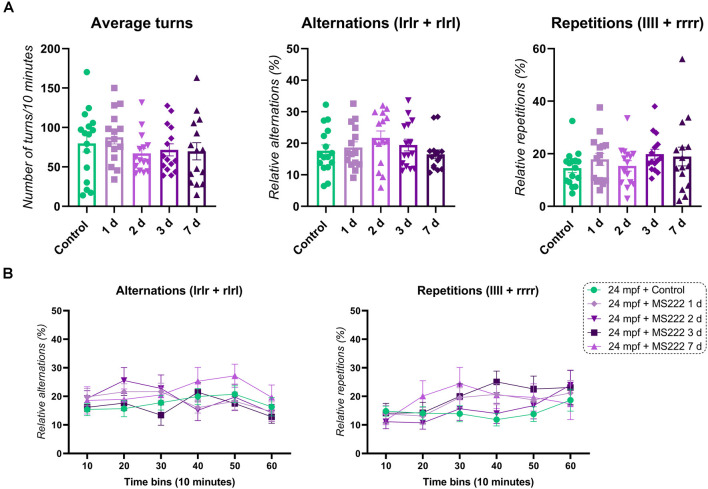
FMP *Y*-maze response of 24 mpf animals 1, 2, 3, or 7 days after exposure to MS222. **(A)** Overall behavioral patterns/10 min. **(B)** Alternations and repetitions across time for 24 mpf controls vs. 1 day vs. 2 days vs. 3 days vs. 7 days after MS222 exposure. Data were represented as mean ± S.E.M and analyzed by one-way ANOVA or two-way repeated measures ANOVA, followed by Tukey’s test multiple comparison test (*n* = 15–16 per group).

Aging fish (24 mpf) when treated with MS222 and tested 1 day or 7 days after exposure did not show significant changes for alternations (*F*_(4, 71)_ = 1.155; *p* = 0.3381), repetitions (*F*_(4, 71)_ = 1.370; *p* = 0.4417) and average turns (*F*_(4, 71)_ = 0.8366; *p* = 0.5065; Figure 2A). For cognitive flexibility, no significant effects for treatment (*F*_(4, 71)_ = 1.155; *p* = 0.3381), time (*F*_(4.554, 323.4)_ = 1.815; *p* = 0.1164) or interaction between factors (*F*_(20, 355)_ = 1.013; *p* = 0.4456) on alternations across time were observed. A significant effect for subject was found (*F*_(71, 355)_ = 2.112; p*** < 0.0001; Figure 2B). Only a significant effect for time (*F*_(4.554, 323.4)_ = 3.529; p*** = 0.0060) and subject (*F*_(71, 355)_ = 2.779; p*** < 0.0001) with no effect for treatment (*F*_(4, 71)_ = 0.9476; *p* = 0. 4417) or interaction (time vs. treatment; *F*_(20, 355)_ = 0.7931; *p* = 0. 7223) was observed for repetitions across time in animals exposed to MS222 (Figure 2B).

### MS222 vs. Age—Interactions Between Drug and Animals’ Age

**Figure 3:**
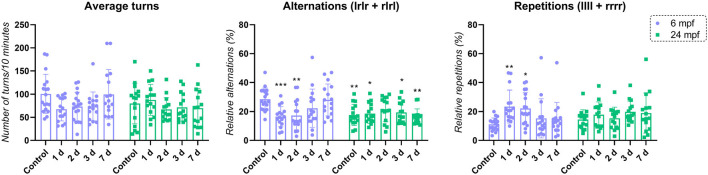
Comparison between age and MS222 effects. Data were represented as mean ± S.E.M and analyzed using two-way ANOVA followed by Tukey’s *post hoc* test. Asterisk represents significant differences compared to control 6 mpf (p* < 0.05; p** < 0.005 and p*** < 0.0005; *n* = 15–18 per group).

Finally, in order to understand the interactions between animals’ age and the exposure to MS222 affecting memory and cognition, two-way ANOVA was performed and a significant effect for interaction (age vs. treatment; *F*_(4, 150)_ = 5.654; p*** = 0.0003) and age (*F*_(1, 150)_ = 7.199; p*** = 0.0081) was observed for alternations. No significant effect of treatment *per se* was observed on the average of alternations (*F*_(4, 150)_ = 2.031; *p* = 0.0929). For repetitions, no significant effect age (*F*_(1, 150)_ = 4.913e-005; *p* = 0.9944) was observed. However, a significant treatment (*F*_(4, 150)_ = 2.723; p*** = 0.0317) and interaction between factors effect (*F*_(4, 150)_ = 2.511; p*** = 0.0442) was observed similarly to the findings described in Figure 1A. The average of turns was not affected having no effect on interaction (age vs. treatment; *F*_(4, 150)_ = 2.110; *p* = 0.0824), treatment (*F*_(4, 150)_ = 1.670; *p* = 0.1599) and age (*F*_(1, 150)_ = 1.813; *p* = 0.1802; Figure 3).

## Discussion

Although there is still a lot of conflicting data regarding the role of anesthetic drugs in inducing memory and cognitive deficits after routine procedures, the majority of the studies support that the effect will mainly depend on the drug, exposure time/concentration, and the individual’s age. Here, we found that MS222 caused an impairment in working memory and cognitive flexibility 1 and 2 days after the exposure, but this was dependent on the age of the animal. Adult (6 months) zebrafish had a significant decrease in their working memory, as demonstrated by them showing a lower number of alternations during the 60 min in the FMP *Y*-maze test. Data across time also suggested that fish exposed to MS222 and tested 1 and 2 days after did not show a linear increase in alternations over the course of the trial, which represents a lack of cognitive flexibility during the test. The exposure to MS222 also resulted in increased repetitions in young adults, which can an indicate increased stress-related response of animals 1 and 2 days after the drug exposure. However, animals tested 3 and 7 days after the exposure had a significant recovery, showing expected baseline parameters for working memory, cognitive flexibility, and abnormal repetitive behavior. In addition, we found that although aging fish showed a decline in the average alternations and alternations across time compared to adult animals (replicating what we have previously reported; Cleal et al., 2021a), we saw no decline in performance following MS222 exposure, independently of the day after exposure, indicating differential effects of the drug on memory and cognition depending on age. The data here shows, for the first time, the short- and long-term effects on memory and cognition of the use of the anesthetic MS222 in zebrafish and will be useful when planning experimental designs using this species in the future.

Although MS222 is one of the major anesthetic agents used in fish species, this drug is often reported as aversive to zebrafish promoting distress in animals when exposed to unbuffered and buffered concentrations of 250 mg/l (Wilson et al., 2009). MS222 is also responsible for the increase of erratic movements during anesthesia (Collymore et al., 2014) however, its effects after routine procedures are virtually unexplored. A recent study investigated a variety of anesthetic drugs in adult zebrafish, including MS222 (175 mg/l), and showed no effects on anxiety an hour after the drug exposure, suggesting full recovery at 13–16 mpf (Jorge et al., 2021). MS222 (168 mg/l) was also shown to have no effects in anxiety and stress-related parameters of adults at ~8 mpf after 30 min of recovery from anesthesia (Nordgreen et al., 2014). Conversely, here, we found that 6 mpf animals had decreased average alternations and increased repetitions in the FMP *Y*-maze, together with disrupted patterns of alternations across time, as animals did not show an alternation peak at 40 min compared to control animals. A previous study that focused on the characterization of the FMP *Y*-maze showed that alternations are the preferred searching strategy used for zebrafish, mice, and humans when navigating the maze (Cleal et al., 2021b). This pattern strongly increases across time, having its peak in zebrafish at the 40 min mark in the test and then gradually decreases. Drugs that affect memory and cognition (e.g., MK-801) significantly affect alternations in the FMP *Y*-maze supporting (Cleal et al., 2021b) the importance of alternations for working memory in healthy animals. Meanwhile, the overall alternations are associated with the animals’ working memory, the analysis of alternations across time provides valuable information about the animals’ cognitive flexibility in this task. Therefore, although some drugs do not affect the animals’ working memory, they can significantly affect cognition across time (Cleal et al., 2021a, b). Altogether, the disrupted patterns of alternations across time found here suggest that zebrafish exposed to MS222 have working memory and cognitive flexibility deficits up to 48 h after exposure.

Exposure to MS222 was also previously shown to block the activity of both sensory and motor nerves in vertebrate species (Ramlochansingh et al., 2014). Although MS222 dynamics in the zebrafish blood-brain barrier are not well-known, this drug was shown to cross the blood-brain barrier through immersion exposure in other freshwater species such as goldfish (*Carassius auratus*) and carp (*Cyprinus carpio*, Hunn, 1970). MS222 is known for blocking voltage-sensitive sodium channels (Frazier and Narahashi, 1975), and in different fish species, this drug has been shown to decrease the activity in the lateral line nerve of oyster toadfish (*Opsanus tau*, Palmer and Mensinger, 2004) and the sensitivity to depolarizing current in dorsal neurons in the cunner (*Tautogolabrus adspersus*, Arnolds et al., 2002). The putative mechanisms in which MS222 may decrease memory and cognition is still unknown since most studies have focused on the drug exposure and later stress-related responses. However, it is known that sodium channel blockers can decrease working memory in young mice due to their deleterious effects on the prelimbic cortex (Vandesquille et al., 2013). Although the effect of sodium blockers decreasing working memory was observed across studies using different behavioral tasks, no effects in the consolidation and retention of memories were linked to blockage of sodium channels (Khakpour-Taleghani et al., 2009). Because here we only investigated the activity of MS222 in zebrafish young adult and aging animals working memory and cognitive flexibility, future works looking at the effects of MS222 should also evaluate other memory functional stages such as consolidation and retention using other behavioral tasks.

Differently from alternations, abnormal repetitions in the FMP *Y*-maze have been found as the main response of zebrafish in front of an acute stressor, the conspecific alarm substance (CAS). The neurobiology of repetitive behaviors is directly related to stress and regularly discussed as a coping mechanism to reduce the arousal level of the animal when it is exposed to stressful events (Langen et al., 2011). Here, we found that animals exposed to MS222 show increased repetitive behavior after 1 and 2 days of the exposure. Although this is indicative of increased stress-related response, as previously discussed, the data regarding anxiety and stress-related phenotypes in zebrafish exposed to MS222 are still conflicting. Thus, the analysis of the mechanisms underlying changes in cognition and stress-related responses are still necessary to understand whether the behavioral patterns observed here are a result of the increased stress response or if there are any other mechanisms involved. Importantly, after 3 and 7 days, zebrafish showed fully recovered behavior with normal patterns of both alternations and repetitions.

In rodents, repeated anesthesia impairs memory and psychomotor performance to a greater extent in aged mice, affecting the animals’ acquisition of a novel motor skill but anesthesia by itself did not lead to any prolonged cognitive impairment in aged animals (Butterfield et al., 2004). Here, in both ages, anesthesia by itself did not induce long-term cognitive deficits; however, aging animals did not show any differences in alternations in the short-term (1, 2 or 3 days after MS222 exposure) or long-term (7 days after MS222 exposure). The lack of effects of MS222 in aging animals may occur based on two hypotheses: (1) MS222 differently modulates memory and cognition in aging animals without causing changes in working memory and cognitive flexibility or (2) the lack of effects of MS222 observed in aged animals was owing to a ceiling effect. Aging is associated with a general deficit in working memory and cognitive flexibility in older fish (Cleal et al., 2021a) and humans (Pliatsikas et al., 2018), and this may have masked the impact of the drug. However, it appears from our data that there is no additive effect of MS222 on existing measures of cognitive decline in older animals.

Although the dose-response effects of MS222 at zebrafish early developmental stages were previously studied (Rombough, 2007), there is no information regarding how adult and aging animals respond to MS222 across the life span. Therefore, further investigation is necessary to understand if the lack of response to MS222 observed in aging animals is due to a difference in drug metabolism or if there is a ceiling effect occurring.

## Conclusions

In conclusion, our data showed that the use of the anesthetic MS222 in routine procedures can significantly affect memory and cognition of adult fish up to 2 days after the exposure. Specifically, the use of MS222 decreases working memory and cognitive flexibility in animals, with 6 months old showing full recovery 3 days after the drug exposure. Abnormal repetitive behavior is also observed in 6 mpf animals exposed to MS222 and tested 1 and 2 days after the drug exposure, which could be a result of increased stress-related response in those animals. Regarding the effects of MS222 in aging fish, no differences for these parameters were observed in 24 mpf zebrafish. Aging plays an important role in cognition and aging animals had lower working memory and did not show cognitive flexibility which could justify the lack of effects of MS222 in aging fish. The data showed here does not only provide valuable relevance in a translational aspect considering that the use of MS222 as an anesthetic drug can affect memory and cognition in other species, but also for those who work with zebrafish and often need to submit fish through routine procedures involving the use of MS222 having further behavioral testing planed in their experimental design.

## Data Availability Statement

The datasets presented in this study can be found in online repositories. The names of the repository/repositories and accession number(s) can be found below: https://osf.io/x6tkz/.

## Ethics Statement

The animal study was reviewed and approved by University of Portsmouth Animal Welfare Ethical Review Board.

## Author Contributions

BF and MP designed the experimental procedures and analyzed the data. MP contributed for reagents/analysis tools. BF and NA performed the experiment. BF, NA, and MP contributed for the manuscript writing. All authors contributed to the article and approved the submitted version.

## Conflict of Interest

The authors declare that the research was conducted in the absence of any commercial or financial relationships that could be construed as a potential conflict of interest.

## Publisher’s Note

All claims expressed in this article are solely those of the authors and do not necessarily represent those of their affiliated organizations, or those of the publisher, the editors and the reviewers. Any product that may be evaluated in this article, or claim that may be made by its manufacturer, is not guaranteed or endorsed by the publisher.
